# Renal mass imaging modalities: does body mass index (BMI) matter?

**DOI:** 10.1007/s11255-024-03962-5

**Published:** 2024-03-18

**Authors:** Young Son, Mark E. Quiring, Raeann M. Dalton, Brian Thomas, Noah Davidson, Dayna DeVincentz, Collin Payne, Sahil H. Parikh, Benjamin A. Fink, Thomas Mueller, Gordon Brown

**Affiliations:** 1 Department of Urology, Jefferson Health New Jersey, 18 E Laurel Rd, Stratford, NJ 08084 USA; 2 Texas College of Osteopathic Medicine, University of North Texas Health Science Center, Fort Worth, TX USA; 3 School of Osteopathic Medicine, Rowan University, Stratford, NJ USA; 4 Lake Erie College of Osteopathic Medicine, Erie, PA USA; 5 College of Science and Mathematics, Rowan University, Glassboro, NJ USA; 6 College of Osteopathic Medicine, Nova Southeastern University, Tampa Bay, FL USA

**Keywords:** Renal mass, Imaging modality, Body mass index, Size estimation, Renal cell carcinoma

## Abstract

**Purpose:**

Accurate measurement of renal mass size is crucial in the management of renal cancer. With the burdensome cost of imaging yet its need for management, a better understanding of the variability among patients when determining mass size remains of urgent importance. Current guidelines on optimal imaging are limited, especially with respect to body mass index (BMI). The aim of this study is to discern which modalities accurately measure renal mass size and whether BMI influences such accuracy.

**Methods:**

A multi-institutional chart review was performed for adult patients undergoing partial or radical nephrectomy between 2018 and 2021, with 236 patients ultimately included. Patients were categorized by BMI (BMI 1: 18.5–24.9, BMI 2: 25–29.9, BMI 3: 30–34.9, and BMI 4: ≥ 35). The greatest mass lengths were compared between the pathology report and the following: computerized tomography (CT), renal ultrasound, and magnetic resonance imaging (MRI).

**Results:**

The difference between greatest length on CT with contrast and MRI were significantly different when compared to pathologic measurement. BMI groups 3 and 4 were found to have a significant difference in size estimates compared to BMI 2 for CT with contrast. No difference was found between size estimates by BMI group for any other imaging modality.

**Conclusion:**

CT with contrast becomes less accurate at estimating mass size for patients with BMI > 30. While contrast-enhanced CT remains a vital imaging modality for tissue enhancement in the context of unknown renal masses, caution must be used for mass size estimation in the obese population.

**Supplementary Information:**

The online version contains supplementary material available at 10.1007/s11255-024-03962-5.

## Introduction

The incidence of renal cell carcinoma (RCC) has been increasing with the use of cross-sectional imaging [1]. In evaluation of renal masses, imaging is an important indicator in determining the most appropriate plan of action. Size is a key variable in the work-up and management of renal masses suspected to be cancerous as there is a known positive correlation between tumor size and malignant potential in RCC [2]. This is reflected in the National Comprehensive Cancer Network (NCCN) guidelines, which recommend active surveillance as an alternative option for T1a tumors [3]. Additionally, current guidelines support the use of imaging until adequate characterization of the mass is obtained, and even recommend supplementary abdominal and chest magnetic resonance imaging (MRI) for suspected lymphovascular invasion [3, 4].

Though optimal preoperative imaging is desired for the planning and execution of an effective operation, there exists an underlying provider preference for imaging that may not be as well-supported as other perioperative guidance. This “carte blanche” prescription often leads to the unnecessary requisition of multiple studies. Currently, renal ultrasound (RUS) is used most often for the initial diagnosis of a renal mass, however, computed tomography (CT) is more commonly used for staging and characterization [5].

While renal mass size is not the sole factor in determining therapeutic approach, it greatly impacts the peri-operative planning for those who ultimately undergo nephrectomy [2, 3]. Given the growing cost of advanced renal imaging, limited access for patients in rural and economically disadvantaged areas, and the limitation of current guidelines on the optimal preoperative modality for RCC, this study aims to improve the management of suspicious renal masses.

## Materials and methods

### Data collection

A retrospective multi-institutional chart review was performed on all patients who presented between January 1, 2018, and December 31, 2021, for partial or complete surgical resection of at least one kidney due to a pre-operative renal mass identification of any size. This included data from three different hospitals and six operating surgeons. Patients were excluded if found to have insufficient demographic or imaging records, or if the suspected renal mass was classified as indeterminate or found to be an angiomyolipoma. Patients were then categorized into four groups based on BMI categories (BMI 1: 18.5–24.9, BMI 2: 25–29.9, BMI 3: 30–34.9, and BMI 4: ≥ 35).

The variables collected from the medical record include the following: age, sex, body mass index (BMI), nephrectomy type (radical or partial), intrarenal mass location (upper, mid, lower), mass description, size on radiologic imaging report (in up to three dimensions), radiologic descriptors, size on pathology report, specimen weight, NCCN cancer stage (when applicable), pathologic diagnosis of mass, and postoperative surveillance imaging modality chosen (if present).

### Definitions

The imaging modalities were defined as CT with contrast (CTwC), CT without contrast (CTwoC), RUS, and MRI with gadolinium. The dimensions reported per radiology reports were in three numerical values. The radiology reports were inconsistent with the use of volumetric equations for the measurement of renal masses. As renal masses are not spherical in size, the reports of the sizes of renal masses were reported with the largest dimension as the first numerical value in succession to the lowest dimension as the last value reported. An identical reporting system was used for specimen reports by the pathologist. BMI was categorized into 4 groups based on the National Institute of Health and World Health Organization classifications. BMI 2 was chosen as the representative group for comparative analysis as this ‘overweight’ classification is the age-standardized mean BMI in North America [6].

### Statistical analysis

The difference between the greatest dimension as reported radiologically and the greatest dimension as reported pathologically was found for each imaging modality. All dimensions and differences between them are reported in millimeters (mm). Negative differences (i.e., imaging size found to be less than pathologic size) are indicated as such using the negative symbol (−). The differences in greatest dimension were compared using a paired t-test for normally distributed data and a Wilcoxon paired test for non-normally distributed data. ANOVA for normal distribution or Kruskal Wallis analysis for non-normal distribution was used for numeric patient demographics. Chi-square was used for categorical patient demographics. Two sample t-test was used to identify if there was a difference in the discrepancy of radiological versus pathological greatest dimension between BMI categories. Statistical significance was accepted at p < 0.05.

## Results

A total of 236 patients were included in the study (Table 1). Of the total cohort, the majority (n, % of total) were classified as BMI 2 (n = 88, 37.3%), followed by BMI 3 (n = 60, 25.4%), BMI 4 (n = 46, 19.5%), and BMI 1 (n = 42, 17.8%). The mean age of the BMI groups was similar within BMI 1, 2, 3, and 4 groups (63.05, 63.35, 63.08, and 62.57 years) with a total cohort age of 63.08 years. BMI 2 had the highest rate of males, consisting of 77.3% of the group, while the highest of females was seen in the BMI 4 group, comprising 50% of the group. The majority of patients underwent partial nephrectomy (150, 63.6%). There was no difference between rates of radical and partial nephrectomy among the BMI groups (p = 0.60). The renal masses were evenly distributed among the upper pole (33.5%), middle pole (33.5%), and lower pole (33.1%).

**Table 1:** Patient demographics and renal mass characteristics

	Total cohort		BMI 1		BMI 2		BMI 3		BMI 4		p value
# of Subjects	236		42	(17.8%)	88	(37.3%)	60	(25.4%)	46	(19.5%)	
Patient demographics											
Mean age (range) in years	63.08	(31–87)	63.05	(36–85)	63.35	(32–84)	63.08	(45–81)	62.57	(31–87)	0.98
Male sex	161	(68.2%)	28	(66.7%)	68	(77.3%)	42	(70.0%)	23	(50.0%)	**0.02**
Nephrectomy											
Radical	86	(36.4%)	13	(31.0%)	31	(35.2%)	26	(43.3%)	16	(34.8%)	0.60
Partial	150	(63.6%)	29	(69.0%)	57	(64.8%)	34	(56.7%)	30	(65.2%)	
Mass location											
Upper pole	79	(33.5%)	16	(38.1%)	32	(36.4%)	18	(30.0%)	13	(28.3%)	0.38
Middle	79	(33.5%)	12	(28.6%)	26	(29.5%)	19	(31.7%)	22	(47.8%)	
Lower pole	78	(33.1%)	14	(33.3%)	30	(34.1%)	23	(38.3%)	11	(23.9%)	

Significant p values are in bold. BMI groups defined as BMI 1: 18.5–24.9, BMI 2: 25–29.9, BMI 3: 30–34.9, and BMI 4: ≥ 35

The difference between the greatest mean dimension of the imaging modalit ies and pathology report was calculated for each BMI group. The most common imaging modality used within the cohort was MRI (n = 115), while CTwoC was the least common (n-41). The results (mean [SD]) are listed by BMI category. BMI 1: CTwC − 0.03 [0.49], CTwoC 0.4 [0.57], MRI 0.18 [0.58], and RUS 0.22 [0.7]. BMI 2: CTwC − 0.03 [0.93], CTwoC − 0.32 [0.74], MRI 0.08 [1.0], and RUS 0.2 [1.2]. BMI 3: CTwC 0.46 [0.52], CTwoC 0.49 [1.01], MRI 0.20 [0.59], and RUS − 0.05 [1.1]. BMI 4: CTwC 0.40 [0.51], CTwoC − 0.38 [0.76], MRI 0.16 [0.61], and RUS − 0.8 [2.4] (Table 2).

**Table 2:** Greatest mean difference, imaging modality versus pathology report

Mean differences (mm) ± SD	CT w/ contrast (n = 80)	CT w/o contrast (n = 41)	MRI (n = 115)	RUS (n = 49)
Total cohort	0.21 ± 0.72	− 0.11 ± 0.85	0.14 ± 0.76	− 0.04 ± 1.45
BMI 1	− 0.03 ± 0.49	0.4 ± 0.57	0.18 ± 0.58	0.22 ± 0.7
BMI 2	− 0.03 ± 0.93	− 0.32 ± 0.74	0.08 ± 1.0	0.2 ± 1.2
BMI 3	0.46 ± 0.52	0.48 ± 1.01	0.2 ± 0.59	− 0.05 ± 1.1
BMI 4	0.4 ± 0.51	− 0.38 ± 0.76	0.16 ± 0.61	− 0.8 ± 2.4
Mean difference (p value)				
Total cohort	**0.01**	0.40	**0.04**	0.54
BMI 1 vs. BMI 2	0.99	0.15	0.61	0.95
BMI 3 vs. BMI 2	**0.02**	0.05	0.52	0.57
BMI 4 vs. BMI 2	**0.04**	0.86	0.70	0.27

Significant p-values are in bold. All values are reported in millimeters (mm). SD = standard deviation. BMI groups are defined as BMI 1: 18.5–24.9, BMI 2: 25–29.9, BMI 3: 30–34.9, and BMI 4: ≥ 35

Among the total cohort, the difference between the greatest mean dimension on CTwC compared to the greatest mean dimension on the pathology report was found to be statistically significant (p = 0.01). A subanalysis of the data showed that 9 (8.3%) of the patients that obtained a CTwC had measurements > 3 cm that showed < 3 cm on pathological measurement, a cutoff commonly used to determine mass management. MRI was also shown to have significant variance within the total cohort (p = 0.04), but this significance was lost once MRI measurements were compared between BMI groups. There was no significant difference found between CTwoC or RUS when compared against the pathology reports.

When compared to the reference group (BMI 2), the greatest mean difference for CTwC for both BMI 3 (0.46 vs − 0.03, p = 0.02) and BMI 4 (0.40 vs − 0.03, p = 0.04) were found to be significantly different (Table 2). All remaining imaging modalities had similar greatest mean differences between the BMI groups.

## Discussion

According to the 2021 American Urological Association (AUA) Guidelines, active surveillance is recommended for solid masses smaller than 2 cm or complex cystic masses every 3–6 months with cross-sectional imaging and/or RUS, or for those with elevated surgical risk or limited life expectancy [7]. The American Society of Clinical Oncology Guidelines also recommend active surveillance for renal masses 4 cm or less with axial abdominal imaging or RUS in similar scenarios, but also lack discussion on the influence of body mass index [8]. The present findings suggest that CT with contrast may remain a consideration for active surveillance in patients with a BMI of 29.9 or less but should be avoided in higher indexes to avoid size overestimation. These findings should also provide confidence to providers who only secondarily choose RUS due to contraindications to CT such as contrast allergy, end-stage renal disease, pregnancy, obscuring artifact, or unable to tolerate a supine position.

For estimating renal mass size, CT with contrast and MRI were found to have significant differences in reporting of greatest renal mass dimension compared to findings on specimen examination. Furthermore, when subgrouped into BMI ranges, CT with contrast was found to overestimate renal mass size in those with a BMI of 30 or greater compared to patients with a BMI of 25–29.9. This diminished accuracy for estimating mass size in the obese population calls into question whether such imaging should be chosen for accurate tumor sizing in this population. CT without contrast and RUS did not show a difference in predicting the size of a renal mass within any BMI cohort, suggesting that equivocal preference should be given when choosing the imaging method for active surveillance of renal masses in similar patients.

The utility of contrast-enhanced CT lies in the increased attenuation to identify malignant masses [9]. Without contrast, most renal masses have attenuation values that approach the surrounding parenchyma, making a radiologic read and caliper measurement more technically challenging. Therefore, CT with contrast should still be used for the initial diagnosis of suspected renal cell carcinoma, regardless of BMI. Once a lesion has been characterized and the active surveillance has been chosen, contrast-enhanced CT may remain beneficial for those with BMI < 29.9 once the risks and benefits of both radiation exposure and iodine contrast administration have been weighed. However, there remains no clearly favored imaging modality for active surveillance, per 2021 AUA Guidelines. In this cohort (BMI < 29.9), it may be appropriate for physicians to use clinical judgment in determining which modality is most appropriate in order to properly utilize the active surveillance phase.

For those with BMI > 29.9, contrast-enhanced CT may overestimate either size or growth rate of the mass during active surveillance, potentially leading to unnecessary treatment. This difference is elucidated by an analysis of our data for a renal mass cutoff of ≤ 3 cm by pathological measurement. While masses in patients with BMI < 29.9 are overestimated in 8.3% of cases, masses in patients with BMI > 29.9 are overestimated in 19.2% of cases. This difference shows that there is an increased chance of overtreatment in the higher BMI cohorts which can significantly impact the quality of life. With new literature providing more information on second-generation gadolinium agents [10], active surveillance for patients with BMI > 29.9 with contrast-enhanced MRI may still be appropriate, even in individuals with chronic kidney disease. Moreover, regardless of BMI status, chest and abdominal MRI with contrast is recommended for those with suspected vena cava infiltration, given the nature of characterizing lesions in this region.

Specific patient populations should also be discussed given the current findings. RUS is most utilized for renal mass imaging in the pediatric and pregnant populations to limit radiation exposure [11, 12]. Estimation of renal mass size may be just as accurate in these cohorts. It is unclear, however, whether the relationship to BMI would show similar results in such groups. Extrapolated from a 2015 meta-analysis regarding prenatal US showed that a BMI > 40 posed multiple challenges including a decreased detection rate of fetal anomalies in the second trimester [13], lending credence to the idea that large volumes of adipose tissue present challenges to US wave penetration. Additionally, RUS is often employed over other modalities for those with end-stage renal disease, as the use of contrast may be contraindicated for glomerular filtration rates less than 30 ml per minute [14]. This patient population, too, may require MRI or RUS if obese.

The primary strength of the current study is that, to our knowledge, this study is the first to use BMI as an independent factor for the visualization of kidney masses with ultrasound with contrast. Limitations of the current study include those inherent to any retrospective, database-driven design. The data included may not be representative of the entire population, making it susceptible to selection bias. Relationships might be impacted by confounding variables that multivariate analysis did not take into consideration. Another weakness of our study is the variability in size reporting for both pathology and radiology reports. This data relied on the radiologists’ and pathologists’ reads, and variability between providers could account for differences seen. It is also possible that masses were not able to be completely resected, again causing a considerable difference between pathology and radiology reports. Ultrasonography is user dependent and could account for an additional limitation. Correct instrument variables including proper transducer, equipment settings, sonographic gel, and technique are crucial in obtaining usable diagnostic images. Along with user variability ultrasound technology can vary wildly from institution to institution with no set technology standard. Further research is needed to isolate if CT imaging with contrast becomes less accurate in patients with BMI ≥ 30 due to intrinsic properties of adipose tissue or some other factor not elucidated by the present study. Additionally, contrast-enhanced ultrasonography may become an important tool for renal mass characterization and should be a focus of future projects, as this may prove non-inferior to CT.

## Conclusion

The current study suggests that BMI does influence the accuracy of renal mass size estimation, and that CT with contrast should be avoided for active surveillance of renal masses in the obese population.

## Supplementary Information

Below is the link to the electronic supplementary material.

Supplementary file1 (DOCX 18 KB)

## Data Availability

The data that support the findings of this study are available from the corresponding author upon reasonable request.
