# Supplementary material for: Renal mass imaging modalities: does body mass index (BMI) matter?
Source: Int Urol Nephrol. 2024 Mar 18;56(8):2483–7. doi: 10.1007/s11255-024-03962-5 (PMC11266215; doi:10.1007/s11255-024-03962-5)
Supplement: Supplementary file 1 — Supplementary file1 (DOCX 18 KB) [file 11255_2024_3962_MOESM1_ESM.docx]

**Table S1: Renal Mass Size, Pathology vs Imaging Modalities (Median & Mean)**

| **Total Cohort** | **Volume (cm^3^)** | **Greatest**  **Dimension (cm)** | | **Smallest**  **Dimension (cm)** | |
| --- | --- | --- | --- | --- | --- |
| **Pathology Report** |  | |  | |  |
| Median | 18.72 | | 3.75 | | 3 |
| Mean±SD | 82.17±231.4 | | 4.51±3.05 | | 3.35±2.22 |
| **CT w/ Contrast** |  | |  | |  |
| Median | 36.61 | | 4.15 | | 3.8 |
| Mean±SD | 187.35±564.49 | | 4.91±3.59 | | 4.39±2.82 |
| **CT w/o Contrast** |  | |  | |  |
| Median | 32 | | 4 | | 3.6 |
| Mean±SD | 149.88±311.64 | | 5.04±3.27 | | 4.46±2.88 |
| **MRI** |  | |  | |  |
| Median | 24.86 | | 3.7 | | 3.3 |
| Mean±SD | 119.47±326.03 | | 4.56±3.20 | | 3.91±2.62 |
| **RUS** |  | |  | |  |
| Median | 12.21 | | 3.3 | | 2.6 |
| Mean±SD | 99.93±296.83 | | 4.25±3.21 | | 3.42±2.54 |

All values are reported in centimeters (cm). SD = standard deviation.

**Table S2: Greatest Dimension of Renal Mass by BMI Category**

| **Median (cm)** | **Pathology Report** | **CT w/ Contrast** | **CT w/o Contrast** | **MRI** | **RUS** |
| --- | --- | --- | --- | --- | --- |
| Total Cohort | 3.75 | 4.15 | 4 | 3.7 | 3.3 |
| BMI 1 | 3.5 | 4.2 | 3.5 | 3.5 | 3.4 |
| BMI 2 | 3.5 | 3.6 | 4 | 3.8 | 3 |
| BMI 3 | 3.85 | 4.45 | 7.15 | 3.5 | 3.7 |
| BMI 4 | 4 | 4.15 | 4.3 | 3.9 | 3.5 |
| **Mean±SD (cm)** |  |  |  |  |  |
| Total Cohort | 4.51±3.05 | 4.91±3.59 | 5.04±3.27 | 4.56±3.20 | 4.25±3.21 |
| BMI 1 | 4.3±2.75 | 5.03±3.07 | 4.43±2.94 | 4.65±3.31 | 4.01±3.24 |
| BMI 2 | 4.03±2.18 | 3.94±2.37 | 4.33±2.56 | 4.05±2.33 | 3.82±2.53 |
| BMI 3 | 4.98±3.85 | 5.46±4.48 | 7.59±4.86 | 5.13±4 | 5.42±4.69 |
| BMI 4 | 5.02±3.45 | 5.4±4 | 4.36±1.74 | 4.75±3.44 | 3.97±1.92 |

All values are reported in centimeters (cm). SD = standard deviation. BMI groups are defined as BMI 1:

18.5-24.9, BMI 2: 25-29.9, BMI 3: 30-34.9, and BMI 4: ≥35.
